# Tutorial for Using Control Systems Engineering to Optimize Adaptive Mobile Health Interventions

**DOI:** 10.2196/jmir.8622

**Published:** 2018-06-28

**Authors:** Eric B Hekler, Daniel E Rivera, Cesar A Martin, Sayali S Phatak, Mohammad T Freigoun, Elizabeth Korinek, Predrag Klasnja, Marc A Adams, Matthew P Buman

**Affiliations:** ^1^ Department of Family Medicine & Public Health University of California, San Diego La Jolla, CA United States; ^2^ School of Nutrition & Health Promotion Arizona State University Phoenix, AZ United States; ^3^ School for Engineering of Matter, Transport, and Energy Ira A Fulton Schools of Engineering Arizona State University Tempe, AZ United States; ^4^ Facultad de Ingenieria en Electricidad y Computacion, Escuela Superior Politecnica del Litoral (ESPOL Polytechnic University) Guayaquil Ecuador; ^5^ Kaiser Permanente Washington Health Research Institute Seattle, WA United States; ^6^ School of Information University of Michigan Ann Arbor, MI United States

**Keywords:** adaptive interventions, mHealth, eHealth, digital health, control systems engineering, behavior change, optimization, multiphase optimization strategy, physical activity, behavioral maintenance

## Abstract

**Background:**

Adaptive behavioral interventions are individualized interventions that vary support based on a person's evolving needs. Digital technologies enable these adaptive interventions to function at scale. Adaptive interventions show great promise for producing better results compared with static interventions related to health outcomes. Our central thesis is that adaptive interventions are more likely to succeed at helping individuals meet and maintain behavioral targets if its elements can be iteratively improved via data-driven testing (ie, optimization). Control systems engineering is a discipline focused on decision making in systems that change over time and has a wealth of methods that could be useful for optimizing adaptive interventions.

**Objective:**

The purpose of this paper was to provide an introductory tutorial on when and what to do when using control systems engineering for designing and optimizing adaptive mobile health (mHealth) behavioral interventions.

**Overview:**

We start with a review of the need for optimization, building on the multiphase optimization strategy (MOST). We then provide an overview of control systems engineering, followed by attributes of problems that are well matched to control engineering. Key steps in the development and optimization of an adaptive intervention from a control engineering perspective are then summarized, with a focus on why, what, and when to do subtasks in each step.

**Implications:**

Control engineering offers exciting opportunities for optimizing individualization and adaptation elements of adaptive interventions. Arguably, the time is now for control systems engineers and behavioral and health scientists to partner to advance interventions that can be individualized, adaptive, and scalable. This tutorial should aid in creating the bridge between these communities.

## Introduction

### Background

Overwhelming evidence suggests health behaviors such as smoking, physical activity (PA), and diet are key to preventing noncommunicable diseases such as many forms of cancer, heart disease, and diabetes [1-4]. Across interventions (eg, human-delivered and community-based), statistically significant changes in health behaviors relative to control can be found, but these differences rarely meet clinical targets such as 10,000 steps/day for PA, particularly when focused on behavioral maintenance [5-7]. Mobile health (mHealth) interventions show promise for promoting behavior change [8], but further work is needed to realize their potential for meeting and maintaining behavioral and clinical targets. To accomplish the goal of meeting and maintaining clinically meaningful targets, many have argued for adaptive mHealth interventions that are individualized and vary the intervention based on an individual’s evolving needs [9-18].

Adaptive interventions are complex interventions [19], which, like static interventions (meaning interventions that are delivered the same way to everyone and do not adjust provision of support over time), often include multiple active ingredient components meant to facilitate behavior change, such as goal setting or problem solving. Adaptive interventions include additional elements [9,11,12]. Since adaptive interventions adjust provision of support over time, an additional element is *decision points*, which are the meaningful windows of time when the selection of intervention type or dose (henceforth labeled intervention option) occurs (eg, daily or monthly). Adaptive interventions also include *tailoring variables*, which are the baseline (eg, demographics) and time-varying information (eg, stress, affect, and weather) that informs intervention option selection at each decision point. Finally, *decision rules* operationalize the adaptation by specifying which intervention option to select at a given decision point based on known information such as tailoring variables.

For example, we have been developing an adaptive PA intervention, *Just Walk,* which includes goal setting, positive reinforcement, and self-monitoring components [20-22]. The end goal for this intervention is to help individuals meet and maintain PA guidelines of 10,000 steps per day by developing an intervention that is responsive to the idiosyncratic and dynamic nature of steps (see Case Study Overview section for more details). *Just Walk* includes a target of daily decision making and, thus, the decision point is each morning. *Just Walk* includes multiple tailoring variables (eg, stress, mood, weather, and self-efficacy) that can be used to inform the decision made at each daily decision point. One intervention component within *Just Walk* is a suggested daily step goal that can be adjusted each day depending on a person’s changing needs. A second intervention component is positive reinforcement for achieving goals, which, in this case, involves provision of points that translate into gift cards. For this component, available points can vary each day, thus enabling a mechanism for increasing motivation to meet a given goal on any given day.

As this example illustrates, there are many elements within this seemingly simple adaptive intervention. The central thesis of this work is that adaptive interventions are more likely to succeed at helping individuals meet and maintain behavioral targets if its elements can be iteratively improved via data-driven testing of the elements. The classic evaluation strategy for behavioral interventions is the randomized controlled trial (RCT). An RCT provides information about whether an intervention package can produce an effect relative to a meaningful comparator (eg, current clinical best practice) but limited information about how, when, where, and for whom each element functions to produce the desired effect. As such, an RCT does not provide sufficient insights for supporting data-driven improvement (also called optimization) of the elements of an adaptive intervention such as *Just Walk*.

Control systems engineering is a field that focuses on decision making in systems that change over time. Control engineering is pervasive (eg, pacemakers, climate control, and robotics) but often goes unnoticed as a hidden technology [23] and to date, has only been minimally applied for use in testing and improving behavioral interventions [24-27]. The methods of control systems engineering are well suited to iteratively improving elements of adaptive interventions for real-world health behavior change. For example, control engineering methods can be used to account for and test the value of multiple tailoring variables simultaneously when selecting interventions and can adapt frequently (eg, every second, minute, hour, and day).

The purpose of this paper was to provide an introductory tutorial on when and what to do when using control systems engineering for designing and optimizing adaptive mHealth behavioral interventions. We start with a review of the need for optimization, building on the multiphase optimization strategy (MOST) [28]. Next, we provide a brief overview of control systems engineering with a particular focus on defining key terms and highlighting the general logic that guides control systems engineering. Following this, we describe attributes of problems that are well matched to control engineering, and then we summarize steps to take to design and optimize an adaptive intervention via control systems engineering. We ground this tutorial in our on-going case study, *Just Walk*.

### Optimization: Unpacking Complex Interventions

In classic RCT’s, all elements are combined into a unified package relative to another package. On the basis of this, limited information about each element, such as the tailoring variables to use for individualization or the decision rules to use for adaptation, is available. If an intervention package produces suboptimal results, it will be difficult, empirically, to localize what elements or interaction between elements could be further improved upon to produce a more potent intervention within RCTs.

Collins et al have been pioneering MOST, which provides structure for thinking about optimization of complex interventions [28,29]. MOST is a comprehensive, principled- engineering-inspired framework for optimizing and evaluating behavioral interventions. The framework includes an RCT to conduct summative evaluations of an optimized complex intervention relative to a meaningful comparator, such as current best practice interventions [30], but also includes other experimental designs for iterative improvement and thus, data-driven optimization of a behavioral intervention. Optimization is accomplished on the basis of optimization criteria. Optimization criteria include measures and clinically meaningful trade-offs such as cost, time, or minimal effectiveness targets, with success (or failure) on each metric determined before running an optimization trial. For example, one optimization criteria could be that each intervention component must be significantly better, statistically, than a comparator, and the entire intervention package must be deliverable for less than US $500. Optimization criteria can also include constraints that limit the actions or feasible ranges of the optimization procedure. For example, an adaptive intervention component cannot make drastic changes such as a large jump in suggested step goals from one day to the next.

The most common optimization trial used in MOST (indeed, sometimes inappropriately labeled a MOST trial) is the use of a factorial or fractional factorial design [31]. This optimization trial can be used to optimize static complex interventions to, for example, eliminate ineffective components [32] or test for interaction effects between components [33] in relation to optimization criteria such as cost-effectiveness [31]. As adaptive interventions include additional elements beyond static interventions, methods are required that can support data-driven optimization of these elements.

One approach for optimizing adaptive interventions is the sequential multiple assignment randomized trial (SMART) [34]. SMART is a method that mimics clinical practice and supports the study of decision rules of adaptation, such as what to do with nonresponders. As clinical visits are often separated by weeks or even months, SMART was designed with relatively infrequent decision points (eg, once every 3 months) as plausible moments of adaptation. Furthermore, SMART can only account for relatively few tailoring variables within a given decision rule. As such, SMART is not well matched to adaptive interventions that monitor multiple tailoring variables simultaneously and with frequent decision points, such as daily, as is the case with *Just Walk*.

There is another emerging method for optimizing adaptive interventions called the microrandomization trial (MRT) [35]. MRT involves randomizing provision of support, not between individuals but, instead, at each decision point. For example, if we used MRT for our *Just Walk* intervention, we could randomize whether a suggested step goal was provided each morning or not to test for each day if a step goal increases steps for that day compared with days without a step goal. There are great opportunities for optimizing adaptive interventions via MRT, particularly when coupled with adaptation strategies that are broadly derived from the computer science method called reinforcement learning (RL) [36]. In particular, MRT and RL are well matched to the emerging intervention class called “just-in-time adaptive interventions,” which provide support during “just-in-time” states, meaning, when a person has the opportunity to engage in a positive behavior (or vulnerability to engage in a negative behavior) and the receptivity to want to be provided support [11].

As demonstrated by our publication record [35], we are supportive of the MRT approach. With that said, we contend that there is great opportunity for taking advantage of the rich history and methods from control engineering when optimizing adaptive mHealth interventions. These methods are complementary for optimizing adaptive interventions that, we argue, should both be part of the repertoire of optimization trial methods that health and behavioral scientists could use for optimizing adaptive interventions, what Almiral et al have called the “optimization toolkit” [37,38]. In the remainder of the paper, we highlight the unique value of control systems engineering for optimizing adaptive interventions.

### Control Systems Engineering Overview

Control engineering has a long history dating back nearly a century and is pervasive (eg, pacemakers, artificial pancreas systems, and supply management) [23]. Control systems engineering focuses on decision making in systems that change over time. An algorithm called a controller defines decision rules (often called policies in control parlance) that attempt to balance mathematical equations related to predicted error, which, in this context is deviation from a desired state. For example, a desired state may be 10,000 steps/day, but if a person currently walks 6000 steps/day, then the error is 4000 steps/day. *Controllers* perform the same task as classic tailoring decision rules [11,12] but with important differences. Classic tailoring uses if-then structures, such as if stage of change=X then Intervention=A; if stage of change=Y then intervention=B [9,10]. Although controllers can use if-then structures, they can use other structures, particularly mathematical equations and optimization algorithms that can account for multiple tailoring variables, intervention options, and responses of the person simultaneously.

One can think of this like accounting. The controller keeps a ledger of measurements. In all controllers, including nonmodel-based controllers such as Proportional- Integral-Derivative (PID) controllers [39,40], this ledger includes measurements of provision of intervention options, called *controlled input variables* in control parlance, and outcome variable(s) (or outputs) that can define how close a system (eg, a person) is in relation to the desired state (error), particularly in response to intervention options. Decisions are made based on the dynamic interrelationship between the intervention options and outcome measure in the past (P portion of a PID controller), present (I portion of a PID controller), or the anticipated rate of change in the future (D; note some controllers only include parts of this such as I or PI controllers). For sake of clarity, we label this class of controller as nonmodel-based controllers.

In more advanced controllers that include a dynamical model, such as model-predictive controllers [41], other variables are also measured, including (1) *inputs*, which include endogenous variables that influence the outcome variables (eg, stress and self-efficacy) and (2) *disturbance variables*, which are exogenous variables (eg, weather) the system cannot control and are not attributes of the person but impact the state of the person and, thus, could influence intervention option selection. In the ledger for model-based controllers, not only are intervention options and outcomes tracked, but their interrelationships are defined via a *dynamical model*, which is like a structural equation model but can model dynamics and incorporate a wider range of response options via difference and differential equations [15]. With this information, model-based controllers can simultaneously monitor a wide array of important issues related to individualization and adaptation, such as variables that are particularly influential (eg, one person’s steps are influenced by stress and another by day of the week [22]) or a person’s changing responsiveness to interventions, such as an intervention option only being useful for a limited time via the novelty effect [42].

Both model and nonmodel-based controllers conduct a series of simulations to predict responses to intervention options in the near and distant future, either based on deviations between the desired state and the state of the system alone (nonmodel-based controllers) or via dynamical models (model-based controllers). These forecasts are used to make decisions. The intervention option predicted to most likely foster movement toward the desired state within prespecified constraints (eg, only small daily changes to step goals allowed) is selected. In contrast, if-then rules require knowledge of the match between tailoring variables and intervention options before specification [43]. This difference means that, relative to if-then structures, mathematical equations can manage more complex decision environments (eg, more tailoring variables and interventions options), can function with limited a priori knowledge about an individual, and can perform when a person’s responses fall outside of expectations and thus, are feasibly more responsive to each individual’s changing needs.

Control engineering includes a wealth of methods for optimizing adaptive interventions by managing and mitigating lack of knowledge related to intervention elements. Lack of knowledge can take various forms from sensor noise (eg, measurement noise when inferring steps [44,45]) to incorrect models (eg, inaccurate predictions). *System identification* is an experimental and analytic suite of methods to generate or validate dynamical models for future predictions, [46-49] or, to put it in more behavioral terms, it can be used for rigorous theory testing. System identification “excites” variance with a person via plausible intervention options to test what happens in different states and contexts of the person over time. For example, if a control engineer wished to generate a dynamical model to understand factors that impact a person’s steps, she may vary a person’s daily suggested step goals in different states, such as different days of the week or when stressed vs not [50]. System identification can occur using both open loop and closed loop experimentation. An open loop experiment is “open loop” because the intervention options that are provided to a person are specified a priori and, thus, a person’s responsivity to the intervention options are *not* taken into account when selecting future intervention options. When a person’s data are taken into account to adjust support, this is called a closed loop experiment. *Dynamical systems modeling* analyzes what occurred following the intervention options over time during different conditions to generate a dynamical model for each person and, ideally, a generic dynamical model structure such as a *semiphysical model*, which is useful across individuals.

Key concepts related to testing controllers are performance and robustness. Performance involves how well the controller can produce the desired effect as efficiently as possible. Robustness involves how well the controller can produce desired performance when issues such as poor measurement, models, or interventions or changing responsivity to interventions arise [43]. It is quite common for controllers with high performance to be less robust vs robust controllers to have poorer performance (eg, take longer to achieve the desired state). As such, a central focus of controller design and testing is to define the right balance between performance and robustness, which can occur via *closed loop experimentation* and *robustness testing* [43,50,51].

A closed loop experiment can be used to test the controller in relation to optimization targets, such as meeting and maintaining PA guidelines. It is closed loop because, like in closed loop system identification, a person’s response to each selected intervention option provided is documented and then taken into account when selecting the next intervention option, thus closing the loop. This type of experiment can include a variety of strategies to test the controller. For example, one could systematically vary providing the predicted optimal vs nonoptimal intervention option to test the controller, if appropriate for the research and intervention. This sort of strategy maps on to the computer science concept of exploring vs exploiting [36]. Exploring involves including some randomness to see what will happen when a predicted nonoptimal option was provided. Exploitation, in contrast, involves using all that is known about a person to select the predicted optimal intervention option. Thus, comparing explore vs exploit options is one way to test controllers, particularly related to performance. In contrast, robustness testing [43] involves examining how well the controller can function when issues such as poor measurement or models arise and, thus, is complementary and often done in tandem with closed loop experimentation.

Within control systems engineering, it is common to use all of these methods (ie, system identification, closed loop experimentation, robustness testing) within a single system or individual. In particular, system identification experiments (ie, theory testing) and closed loop experiments (ie, testing with controller actions present to support, among other things, testing of the controller) can be offered sequentially to a single person and, indeed, decision rules can be defined on when to switch from one method to the next. For example, a closed loop experiment might be used to test a controller striving toward helping a person to meet PA guidelines. If the person meets the behavioral target for a prespecified time (eg, 2 weeks), this could trigger the switch to different optimization criteria, such as targeting maintenance of steps and minimization of interactions between the intervention and person (ie, a second controller optimization algorithm for maintenance). The combined study that includes system identification, closed loop experimentation, and robustness testing is what we call a *control optimization trial* that can balance the competing demands of performance (eg, quickly helping a person meet goals) vs robustness (eg, being responsive to individual differences and changing needs). These methods enable a rigorous and efficient approach to optimize elements of an adaptive intervention for each individual.

## Attributes of Problems That Are Well-Suited to Control Systems Engineering

In this section, we describe attributes of problems that are well-matched to control engineering. We ground our discussion within the concrete case study of *Just Walk*.

### Case Study Overview: Just Walk

Convincing evidence indicates PA is valuable for reducing risk of certain types of cancer [52,53], cardiovascular disease [54], and for improving glycemic control [55]. Walking or taking steps is important for all adults but in particular, those who are sedentary, overweight or obese, and in the age range of 40 to 65 years because they are at increased risk of chronic diseases and because this group can safely walk and fit it into their lives [56,57]. With an aging population, step interventions could help prevent chronic diseases, reduce health care costs, and improve functional life years and quality of life [52-55,58-70]. Guidelines for steps suggest 7100 to 10,000 steps/day [56,57], but only one-third of this group meet the guidelines [71-81]. Across PA interventions for older adults (eg, human-delivered and digital), results show 620 steps/day increases, which translate to individuals walking, on average, 5388 steps/day, which is below guidelines [5]. Findings are similar among healthy adults with 496 steps/day achieved, and even high-impact interventions peak at 1363 steps/day increases; both below guidelines [6]. Even among interventions that produce an effect, maintenance is rarely measured and, when it is, it is not achieved for a large number of participants [82-85]. There is a strong need for interventions that can help individuals meet and maintain PA guidelines.

One reason meeting and maintaining PA is hard may be because of the idiosyncratic and dynamic nature of steps. Specifically, taking steps occurs in both incidental and purposeful ways [86-88] such as commuting, leisure walking, or sports and is engaged in differently by different people. Furthermore, when and where individuals fit steps in also changes (eg, weekend warriors vs evening gym rats) over time and also can vary between individuals. Our prior work [22,89] shows that individual variables (eg, stress and busyness) and contextual factors (eg, weekend or weekday) have different relationships to steps for different people. These idiosyncratic determinants change over time. Walking routines change based on a variety of factors such as small stressors (eg, pressing deadlines) to large ones (eg, changing careers and retirement) and context changes (eg, changes in season) [71-73,75,76,80]. It is also common that interventions lose their potency, thus suggesting reduced responsivity [8,84,90,91].

**Figure 1:**
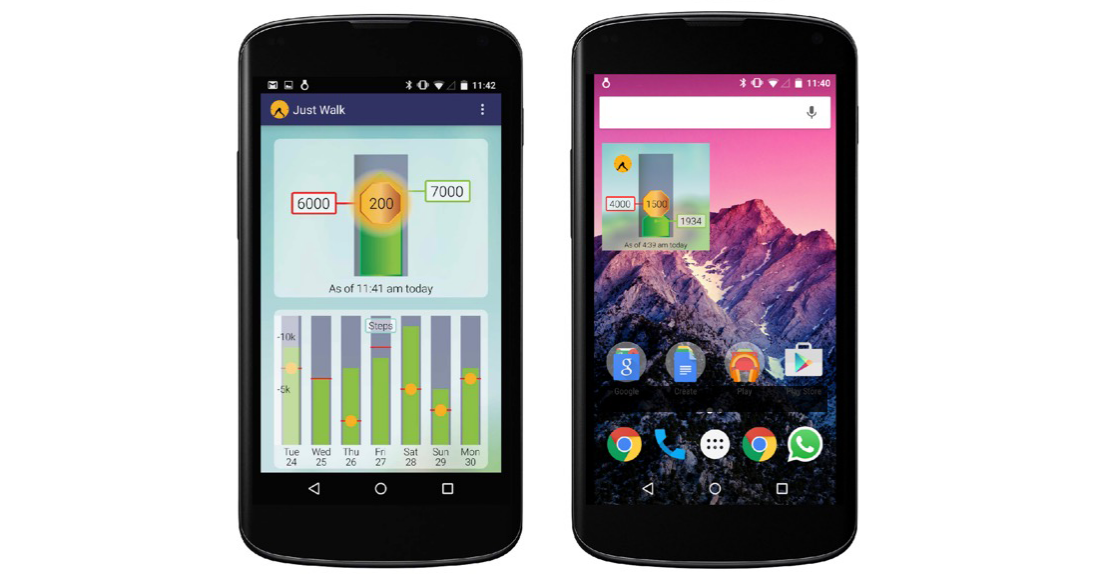
Screenshots of the Just Walk App. The image on the left is the view inside the app, which includes the suggested step goal for the day (in the red box), available points (in gold medal in the middle) and current steps (in green box). Below is the person’s step history. The image on the right is the app’s “widget,” which enables a person to receive feedback relative to their goal without opening the app.

We have been developing *Just Walk* as an mHealth adaptive walking intervention, specifically to account for the inherently idiosyncratic and dynamic nature of walking behavior. Our intervention includes individualized step goal suggestions, self-monitoring (measured via a wearable device), and contingent reinforcement (ie, points and gift cards) that are provided when daily goals are met. In addition, we will supplement our behavior change active ingredients via a range of engagement-supporting tools such as suggestions for weather-appropriate ways to be active. The mHealth system includes, at present, a front-end mobile phone app (Figure 1), a back-end server, and integration with wearable devices (eg, Fitbit) to objectively measure PA.

### Problems That Are Well-Suited to Control Systems Engineering

We turn to a discussion on the types of problems that are well suited for control engineering (see Textboxes 1 and 2). We discuss each below and will use *Just Walk* to illustrate.

First, the problem is dynamic, meaning the input and output variables interact over time. Within *Just Walk*, steps/day is dynamic as it often fluctuates day to day for each person. The factors that impact how many steps a person takes, such as internal states such as stress, busyness, perceived self-efficacy and external states such as weather, also change over time. Any self-regulatory process that can be measured frequently, such as blood pressure, weight, emotion regulation, or glucose regulation within the body, are dynamic and thus, feasibly appropriate for control engineering. Conversely, if the behavioral or clinical target used to define the problem changes slowly (eg, atherosclerotic plague formation, mortality), then control engineering is not appropriate.

Second, interventions are available to foster movement from a less desirable to a more desirable state. As part of this, there are concrete decisions that can be made for each decision point. Note that these decisions can include providing or not providing an intervention or more continuous intervention options (suggested daily step goal).

Within *Just Walk*, the two dynamic interventions are based on Operant Theory [92,93] and the Social Cognitive Theory (SCT) [94]; specifically, the logic of the feedback loop between antecedents, behaviors, and consequences. Within *Just Walk,* the antecedent is a suggested daily step goal, the behavior is steps/day, and the consequence is daily points, which translate into Amazon gift cards. We chose these two dynamic interventions based on past research suggesting that they can influence steps [95,96]. Conversely, if the behavioral or clinical interventions are not particularly dynamic (eg, taking a vaccine that only occurs once) or do not repeat frequently (eg, attempts to facilitate taking a flu vaccine 1x per year), then control engineering is not appropriate.

Third, the target outcome can be measured with sufficient temporal density over an extended period. In the *Just Walk* example, this requirement is met via the use of wearable sensors to track steps. This requirement is available for many of the processes listed above, such as blood pressure, weight, or glucose regulation, along with behavioral targets such as sleep and some forms of diet (eg, chewing as inferred from accelerometry). When there is a lack of a variable that can be measured repeatedly over time, then control engineering methods become less relevant. For example, lack of quality cancer risk metrics, at present, reduces the utility of control engineering for cancer prevention, except for meaningful proximal predictors such as weight or PA for some forms of cancer (at least until more proximal markers of cancer risk can be developed).

Finally, there is a need for definable desirable states for the target outcome(s), which are called *set-points* in control parlance. This is particularly important as it establishes a within-person benchmark of success for the controller and, thus, the optimization criteria for individualization (via tailoring variable selection) and adaptation (via the decision rule). It is important to note that multiple phases, which are labeled states [97] in control engineering to acknowledge the movement between states rather than to imply progression, can be defined, and each state can have its own optimization criteria. Furthermore, multiple levels of success can be defined.

Within *Just Walk*, there are two states: behavioral initiation and maintenance. Our set-point for behavioral initiation is 7100 to 10,000 steps/day based on past work [56,57]. *Just Walk* strives for either 10,000 steps/day per week or, if a person does not seem capable of meeting that goal (ie, a person starts at low steps/day and does not achieve 10,000 steps per day within 6 months), then 3000 steps/day above the person’s baseline median steps is used as the set-point (which usually equates to at least 7000 steps). Within the maintenance state, these set-points are used but with added constraints. During initiation, there is a bias toward providing support, unless a person appears to be responding negatively to the intervention (eg, reduced adherence). In maintenance, *Just Walk* switches toward reducing the total number of interactions, with the ideal of no support provided when not needed. *Just Walk*, thus, does not end but, instead, adapts based on perpetual need, which, conceptually could be a highly cost-efficient approach. With the optimization criteria defined, it enables data-driven optimization for individualization and adaptation.

**Required attributes of a problem that are well matched to control engineering.**

- Dynamic, input-outputs
- Intervention options are available
- Outcome variables are measurable (or inferable) intensively
- A meaningful target or “state” exist

**Desirable attributes of a problem that are well matched to control engineering.**

- Frequent decision points
- Previous theory available to guide model development
- Other feasibly important variables can be intensively measured
- Theorized dynamic interrelationships between inputs or outputs (eg, feedback)

Beyond these requirements, there are several desirable attributes. First, it is advantageous to have frequent decision points, such as every hour, day, or week. Within *Just Walk*, we used a daily timescale. These daily decision points enabled the design of an efficient 12-week study (described below and [98]). Technically, it is possible to develop dynamical models with less frequent decision points; thus, control engineering can be used for stepped care decision making [99]. This longer timescale, however, establishes the need for longer system identification studies. If, for example, we had used a weekly timescale in *Just Walk*, the study would have needed to be 7-times longer. Determination of the appropriate timescale and minimal number of decision points or observations needed can be achieved using simulation studies, such as the ones we conducted for our *Just Walk* study [15,100].

Second, it is desirable if previous knowledge about the phenomenon is available. Within our example, we used the SCT to inform measurement selection, a model structure for defining our dynamical model and the interrelationships between variables, intervention selection, and the design of our study, discussed below [100,101].

Finally, it is desirable that other variables that could impact the outcome can be measured. Within *Just Walk*, we could infer variables passively, such as weather, and ask participants to complete surveys daily with minimal burden [21].

Finally, if there are strong theoretical reasons to hypothesize feedback loops and lagged effects [102], then the suite of methods used by control engineers might be beneficial. This is because dynamical modeling can mathematically specify and thus model and account for issues such as carryover effects, lagged effects, delayed effects, or feedback loops via the use of difference and differential equations [83]. As delineated by SCT, there are multiple theorized feedback loops that can be modeled via dynamical modeling.

## Steps to Take When Using Control Engineering for Adaptive Interventions

### Overview

In this section, we highlight suggested steps that could be used when using control engineering methods to optimize adaptive interventions. A full review on exactly how to do each step is beyond the scope of this introductory tutorial. Instead, for each step, we define *why* the step is important, *what* specific tasks are involved in the steps, and *when* to do the step vs possibly skip the whole step or at least some tasks of the step. To provide insights on *how* to do these steps, relevant references are provided. Each step is grounded with the concrete example of our *Just Walk* intervention.

Although the use of the word “step” may imply a linear process, it often is not. For example, it can be highly advantageous to select a general theoretical model (a task within step 1) and to then define optimization criteria (a task in step 4) before moving on to creating or selecting intervention options (step 2) or to even start with optimization criteria as a definition of success, which is advocated for in agile science [103]. In line with our focus on optimizing elements of an adaptive intervention, essential to this overall process is the use of the iterative process and triangulation of methods to clarify one aspect of the adaptive intervention and then examine its impact on other aspects (see Discussion).

An important prestep is to make an initial decision on the type of controller one is targeting. Although there are many considerations involved in the selection of the appropriate controller, at a high level, selection of one controller over another largely hinges on the anticipated complexity of the dynamical system, the degree to which a model can be generated that is actually predictive or useful for making decisions based on forecasted responses, and the degree to which the dynamics can be inferred from the dynamics of one (or a relatively few number) of variables (nonmodel-based), as opposed to the response of multiple interrelated variables (model-based). If the guiding theoretical model implies a complex dynamic system that would not be well represented by monitoring only intervention options and outcomes, then a model-driven controller would likely be most appropriate. If, however, the dynamics can be picked up adequately with intervention options and outcomes, such as the direction a boat is pointed as measured via a compass as used within a boat autopilot (a classic PID controller), then a nonmodel-based controller is appropriate. There is a lot more subtlety involved in selecting the right controller (eg, the possibility of model-based PID controllers), and interested readers can gain more insights on control options here [39,40,104]. On the basis of the complexity of behavior, we anticipate that it will almost always be best to use model-based controllers. As such, the steps below are suited for model-based controllers.

Suggested steps include the following: (1) derive a preliminary dynamical model; (2) select intervention options (ie, type, frequency, and dosages) and outcomes; (3) conduct system identification (ie, theory testing); (4) design the controller; and (5) conduct a control optimization trial (ie, intervention element testing).

### Step 1: Derive a Dynamical Model

This step is important for establishing a well-specified framework for understanding the eventual adaptive intervention and guiding all subsequent work. The tasks involve first specifying a general theoretical model for guiding the work, then translating that into a dynamical model, and finally, the option of vetting this dynamical model either via simulation studies, secondary data analyses, or both.

Like in MOST, a theoretical model is used to provide structure and specification about key intervention options, outcome measures, and other variables that impact the outcome measures. It is strongly advised to almost always engage in this step as it provides the foundation for understanding predictions and decisions made within the eventual adaptive intervention. The one caveat is when an adaptive intervention is being generated when very little is known about the phenomenon, except that it is highly dynamic. When this is the case, it is often more appropriate to do noninterventional work such as conducting more naturalistic studies such as ecological momentary assessment or human-centered design work [105]. Nonetheless, we could imagine the small possibility of the need to use experimentation to gain insights about a system that is not known. This should only be done when the intervention can be done safely.

Although there is not a single way to develop a theoretical model, we suggest thinking clearly through three reference points and using each to triangulate toward, first, a theoretical model and then a dynamical model. These three references are (1) prior theories, particularly those that have been well-validated in the literature among the target group; (2) prior empirical work about what works in general and other key variables to be aware of for the target group; and (3) hands-on experience and interactions with the target group in the form of human-centered design methods such as interviews, observation, codesign, or prototyping, to gain insights about your target group that may not be well understood or encapsulated in prior theories or evidence. For details on exactly how to create or select an appropriate theoretical model, we suggested the following references (see [22,37,106]).

Translation of a theoretical model into a dynamical model requires far clearer specification of the prediction. For model-based controllers, this task is required. Creating a dynamical model involves clear specification of a variety of issues such as model structure, anticipated directionality and strength of relations between variables, and anticipated dynamics of the interrelationships [97]. For more details on how to do this step, see prior work [97,107,108].

The final optional task within this step is simulation studies or analyses with secondary data to vet a dynamical model. As the previous step highlights, dynamical models often require a high degree of mathematical specification on predictions. The use of simulations, such as changing one variable to see how the other variables might respond within the system, is valuable to gauge if the dynamical model is producing the sorts of effects that would be anticipated. If the simulated changes in one variable produce effects that are not anticipated, this can be used to either check the math or check the assumptions about the problem. Either way, it improves precision and understanding on what is being hypothesized dynamically. Secondary analyses can also be valuable as data can be used to ground the predictions of the dynamical model, again, to see if the dynamical model is working according to both expectations and available evidence. For more details on how to create a dynamical model and do preliminary vetting via simulation studies and secondary analyses, see [15,97,100].

**Figure 2:**
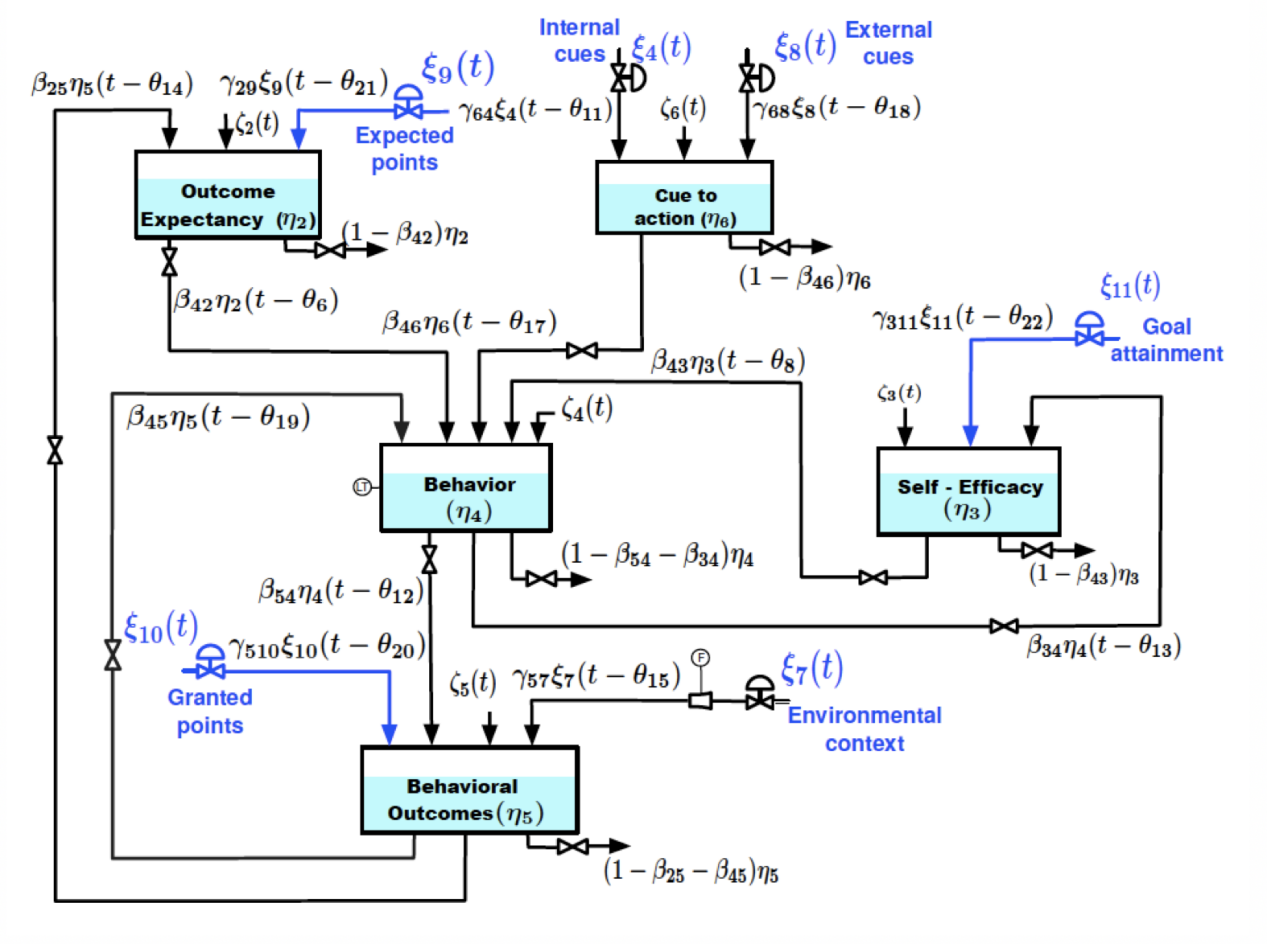
Simplified dynamical model version of Social Cognitive Theory.

Within our *Kust Walk* example, we chose to use prior theories, Operant Theory and SCT, as one foundation for our adaptive intervention. This is based on extensive prior work illustrating the value of these related theories for supporting robust interventions among our target group. We also have ample experience working with our target group for supporting PA via interventions based on them [109] and thus have prior evidence and user interactions. As such, we grounded our model selection based on the three references of prior theory, evidence, and insights from our target group. As this example also suggests, we hypothesized that the dynamics for understanding steps would be best understood using a model-based controller as opposed to nonmodel-based controllers. On the basis of this, we translated SCT into a dynamical model, with full steps and details about this process described in Riley et al [15] and Martin et al [100]. We identified key variables (eg, self-efficacy and outcome expectancies), defined a model structure (Figure 2), and then specified, mathematically, the anticipated interrelationships between variables. After specifying these attributes of the dynamical model, we then ran a series of simulations of theoretical predictions to stress test the model with known psychological concepts such as habituation [15]. We also conducted secondary analyses from prior available evidence [110,111]. We decided on a simplified version of the SCT as a dynamical model to guide the rest of the process, based partially on the results of the simulation studies and secondary analyses.

### Step 2: Defining Interventions Options and Outcomes

Defining target intervention options and outcome metrics are the defining features of an adaptive intervention and, thus, this step is essential. The key tasks of this step include defining the outcome metric(s) being targeted (which will be translated into optimization criteria in step 4), defining the intervention options and then, optionally, also specifying clear dynamic hypotheses on how these intervention options will dynamically interact with the person to produce desired changes to the outcome(s).

Clearly defining the outcomes is a logical follow-up step from the theory and dynamical modeling work. This is because, within the prior step, it is technically possible to do most of step 1, save the secondary analyses, without any concrete outcome measure defined (eg, steps/day or hours of sleep per night). Defining outcome metrics to target is important as it establishes a grounding on the purpose of the specific adaptive intervention. As discussed in the previous section, outcome metrics are best when they can be measured repeatedly over time to establish the current state of the target person relative to the desired final state. The intervention options can then be defined to impact the outcome metrics dynamically. These intervention options could be thought of as the essential levers the adaptive intervention can use to make adjustments and thus, facilitate movement from a less desirable to a more desirable state.

Although not required, it can be valuable to generate a dynamic hypothesis about the interrelationship between an intervention and an outcome to further ground thinking about the intervention. Although there are many ways to think about dynamic hypotheses, one way is to think in terms of outcome responsivity to the intervention options when a person is in a different state or context, including changing disease state or changes in their readiness for change (eg, stages of change). The Transtheoretical Model (TTM) establishes a basic (albeit slow) dynamic hypothesis in that different processes of change are hypothesized to be needed for different stages of change [112,113].

Similarly, one might theorize that a person will respond differently to an intervention if it is provided to them when they are stressed vs not, or at home vs at work. These variations, which are further described in detail elsewhere using the modeling logic of state-spaces [97], provide a structure for thinking through the dynamic interrelationships between interventions, outcomes, and changes in the person and context over time.

These dynamic hypotheses can take various forms. A relatively simple dynamic hypothesis could be to specify if-then statements for different states or contexts of the individual, which is the implicit structure used in the TTM (eg, if stage of change=X then process of change=Y). This could be useful for stepped care interventions (eg, see [99,114-116]). As discussed earlier, control engineering uses mathematical equations for prediction to support dynamic decision making. On the basis of this, dynamic hypotheses are not required to conform to if-then statements, but instead can be defined more mathematically related to predicted changes in key variables. This latter more complex structure is what we use within *Just Walk*.

**Figure 3:**
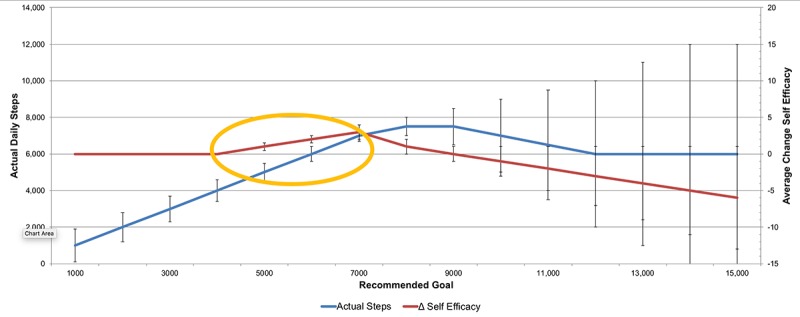
Dynamic hypothesis.

We chose to focus on individualized step goal suggestions and provision of points as our two dynamic intervention components (grounded in self-monitoring as the third, but we assumed that to be a constant intervention component). Our key outcome measure in *Just Walk* is steps per day, as measured via a wearable device. To help define our eventual controller design (step 4), we postulated a dynamic hypothesis that can be specified mathematically but not as an if-then structure. A common hallmark of goal setting includes strategies that help a person define what might be called an *ambitious but doable* target [117]. Within *Just Walk*, we have encapsulated this mathematically as a dynamic hypothesis that is influenced by suggested goals and points (see Figure 3). The figure is meant to visualize the dynamic interrelationship between recommended step goals (x-axis), actual steps taken (y-axis, left side), and the impact on self-efficacy, on average (y-axis, right side). The yellow circle is the hypothesized *ambitious but doable* recommended step goal range that is hypothesized to be optimal for fostering increases over time in self-efficacy. Below this range, and any time a person meets their step goals, we hypothesize will not impact their self-efficacy. Above this dynamic range and we hypothesize that, on average, the person will not attain goals as regularly and, thus, with particularly high goals, will result in an, on average, reduction in self-efficacy. For the person/day represented in Figure 3, if a person’s goal is below 4500 steps, we hypothesize no change in self-efficacy when the goal is attained. If the goal is too high, we expect goal attainment to happen less frequently, on average, which would result in an overall reduction in self-efficacy over time.

For example, if a goal of 9000 steps or greater is suggested for our Figure 3 example, goal attainment will occur less often, on average, resulting in a progressive reduction in self-efficacy as a person reduces their confidence that they can meet challenging step goals. Finally, as self-efficacy and individual and contextual factors change (eg, stress and weather), along with available points, so will the target range (eg, blue line moving up or down). This dynamic hypothesis conforms with the SCT, the rationale of graded goals, and with previous evidence suggesting high goals (ie, 10,000 steps, arguably ambitious for sedentary individuals) resulted in more variable steps [96].

### Step 3: Conduct a System Identification Experiment (That is, Theory Testing)

This step is most distinct from other forms of testing and optimization within behavioral interventions. As such, we include a great deal more information here to highlight the logic and overall approach.

From a control systems perspective, the primary goal of this step is to estimate and validate dynamical models. This is valuable in and of itself, regardless of any subsequent controller, because it is explicitly focused on understanding a “system” or phenomena, such as, in this case, an individual human. To put it in more behavioral terms, system identification is a form of dynamic theory testing. System identification is also important for later steps, if there is a desire to use model-based controllers. Although not commonly the focus in control engineering, this type of study can also be used to select tailoring variables for individuals (ie, data-driven individualization), test dynamic hypotheses, or develop a benchmark comparator for optimization criteria when prior work provides limited insights on a meaningful benchmark. It is also technically possible to conduct a rigorous system identification experiment while also pilot testing aspects of the intervention and other protocols to test feasibility issues, if needed, because system identification is an inherently n-of-1 or idiographic approach, though this last strategy is not necessarily recommended.

As a reminder, within a *system identification* experiment, excitation of variance (ie, providing different intervention options) is provided to the system (in this case, a person) to test what happens in different states and contexts of the system over time. *Dynamical systems modeling* analyzes what occurred following the intervention options over time during different conditions to generate a dynamical model for each person in a scalable fashion. System identification can occur using both open loop and closed loop experimentation but, from the perspective of system identification, these variations are used to validate the dynamical models (ie, theory testing) as opposed to testing the controller (ie, decision rule testing), which is the emphasis in step 5.

Suggested tasks that could be included in this step include the following: (1) design of the system identification experiment and analytic plan and (2) data analyses. If pilot testing of the technology is also needed, other optional tasks could include the following: (1) human-centered design work [103,106,118] to facilitate creation of a useful and usable intervention and (2) creation or selection of the technology tools needed to conduct the intervention (eg, digital health intervention platform). These optional tasks should be conducted when no prior adaptive intervention is available, but otherwise should be skipped as this step is primarily focused on theory testing, not pilot testing. If these optional tasks are conducted, current best practices for human-centered design and feasibility testing should occur [37,103,106,118,119]. The system identification experiment should be conducted when there is inadequate secondary data or theory (often thought of as first principles in control parlance) available about the topic to generate robust dynamical models, when the research question is clearly about dynamic interrelationships within a person (ie, theory testing), or when there is a clear dynamic hypothesis to test about the system or person. The system identification experiment does not necessarily need to be done if the targeted controller is not model-based, such as some forms of PID (and their derivations) controllers [39,40].

In terms of system identification, there is a rich literature, including toolkits within MATLAB (MathWorks), on procedures and best practices for the design of a system identification experiment and analytic plans [47,48]. As a system identification experiment excites variance within a system, the study design involves carefully defining intervention options with a particular eye toward having variability of excitation. Excitation can occur both by varying the amplitude of the differences between intervention options and also the repeatability of intervention options. For example, in our *Just Walk* study, we chose meaningful ranges in terms of the “dosages,” from baseline median steps to double a person’s baseline median steps for step goals and providing between 100 to 500 points for the reinforcement component, with 500=US $ 1. Note also that system identification experiments can include binary intervention options as well (eg, provision of support or not) [120].

With a sense of the amplitude defined, the next task involves designing for adequate excitation over time, which minimizes error for model estimation. This involves the length of a “cycle” and the number of cycles needed in a study to achieve sufficient minimization of error in estimation and validation. A cycle is a deterministic, repeatable pattern that defines provision of intervention options to an individual. Intervention options can be provisioned to mimic randomness via pseudorandom signals that can achieve the valuable properties of randomness for causal inference, while still being deterministic and, thus, repeatable (for more details see [121]). The primary purpose of a cycle is to enable both estimation and validation of dynamical models in terms of their predictive capacity across cycles. Within our *Just Walk,* it was determined that five 16-day cycles would produce sufficient excitation over time to minimize error in estimation and validation with our two interventions delivered orthogonally (discussed in greater detail below on how this was determined [121]). As a side note, it is possible to do estimation and validation with purely random signals, but pseudorandom cycles facilitate aspects of model validation [120,122,123]. The design of a system identification experiment can be done with a number of different toolkits that support simulation of estimation and validation based on different sources of noise or variance in the model [47].

Once data are collected, the process of data analyses takes place. A central logic of dynamical systems modeling, as with other aspects of control systems engineering, is triangulation. In particular, system identification toolkits (eg, those available via MATLAB) include a wide range of strategies to examine time series data produced from system identification experiments, such as different visualizations, step-response curves (ie, the unique influence of each variable on the outcome, much like a partial r2), or model fits for both estimation and validation. Each one of these provides a different understanding on the overall reliability and validity of the dynamical models produced. As such, they are all used with the goal of defining dynamical models that work according to expectations across these tests.

Beyond the criteria used to evaluate the models, there are also different analytic techniques that can be used as part of dynamical systems modeling. For the sake of simplicity, we describe black-box dynamical modeling vs semiphysical or grey-box modeling. Central to these different modeling efforts is the degree to which prior theory and evidence is taken into account when defining a dynamical model structure. On one end are black box models from methods such as Auto-regressive model with eXogenous Input (ARX) modeling, which are much like generalized linear models. These models include no model structure to define the interrelationships between structures beyond ordinary linear regression accounting for repeated measures. Semiphysical modeling, on the other hand, includes theorized model structures, predicted dynamics, and other factors that are either known or theorized to be true in terms of the interrelationship between variables. One could think of semiphysical modeling as a dynamical version of structural equation modeling [22,89]. In brief, theorized model structures, such as Figure 2, along with predicted dynamics (eg, feedback loops are the ways in which the relationships occur dynamically) are articulated within a mathematical model [97,124]. These models can then be compared with the initial black box models on a variety of criteria related to reliability and validity of the models for each person, such as overall model fit, which provides insights on the percentage of variance explained by each dynamical model (for more details see Study Design). This process, thus, enables a rigorous strategy for iteratively developing models of progressively improved predictive capacity for each person, while simultaneously enabling incremental theory testing. Furthermore, particularly related to theory testing, generic model structures can be defined if they prove reliable across individuals, thus providing a structure for translating insights drawn about an individual to be generalized to other individuals and also more generic theory testing and development that is grounded first in individuals rather than starting first in the aggregate.

The final task is to define *good enough* predictive capacity to establish an optimization criterion. If little to no information about what is good enough is available, the above strategy of comparing data-driven vs theory-driven models is a good start. If, however, other parameters or benchmarks are available and meaningful from the literature relevant for the problem domain, then those can be used as starting benchmarks on factors such as model fit. When good enough predictive capacity is reliably being shown across individuals (or at least a large enough portion of individuals, which also can and should be defined), this establishes justification for the development (step 4) and testing (step 5) of a model-driven controller. If not, a nonmodel-based controller could be explored, or the team should examine earlier steps in the process or other optimization trials (eg, between-person factorial trials, SMART, or MRT).

With these tasks defined in abstract, we turn to the *Just Walk* example. In our previous work [21,22,89], we conducted human-centered design work to develop an app for adults who are midlife and older, overweight, and sedentary. We then conducted a 12-week system identification open loop experiment, which is described below. In this context, because we did not have a previous platform, we decided to do the optional feasibility work. For the feasibility aim, this study design could be thought of as a modified variation of a single case experimental design, particularly an ABBBBB trial design with the “A” representing the baseline phase and each “B” representing an intervention cycle that was repeated five times [21]. This design supports testing feasibility issues including limited efficacy, which is defined as within-person changes in steps. Our results suggest (1) our intervention increased steps; (2) good demand, acceptability, implementation, and practicality; and (3) our system identification experiment produces valuable data for dynamical models (for more details see [21]).

To support the eventual controller, we chose to run an open loop system identification experiment. This was because, although we did conduct secondary data analyses to vet our dynamical model [100], the secondary data analyses did not enable us to do rigorous estimation and validation of our dynamical model. On the basis of this, and our desire to develop an eventual model-based controller, we conducted an open loop system identification experiment [89,121,125].

In *Just Walk*, we devised our open loop system identification experiment to estimate and validate our simplified SCT dynamical model (Figure 2) and to test an approach for individualized tailoring variable selection [15,22,100]. As implied by our dynamic hypothesis (see step 2), from an excitation standpoint, this hypothesis requires that we include goals that are *doable*, *ambitious but doable*, and *too ambitious* for individuals. Furthermore, as the hypothesis includes specification that individual differences (eg, stress and busyness) and context (eg, weekday or weekend and weather) that could feasibly influence what is ambitious but doable on any given day for a person, it also established the requirement of repeated observations that are in the three broad category ranges within different states of the individual (eg, high vs low stress) and contexts (eg, weekdays vs weekends) for tailoring variable selection purposes. As states and context cannot be randomized, we instead chose to run the experiment over a 12-week period to increase the likelihood of observing variations in these individual and contextual factors in relation to different suggested step goals and for excitation purposes [121]. Beyond this, expected points was also hypothesized to interact with these other factors and thus varied over time.

**Figure 4:**
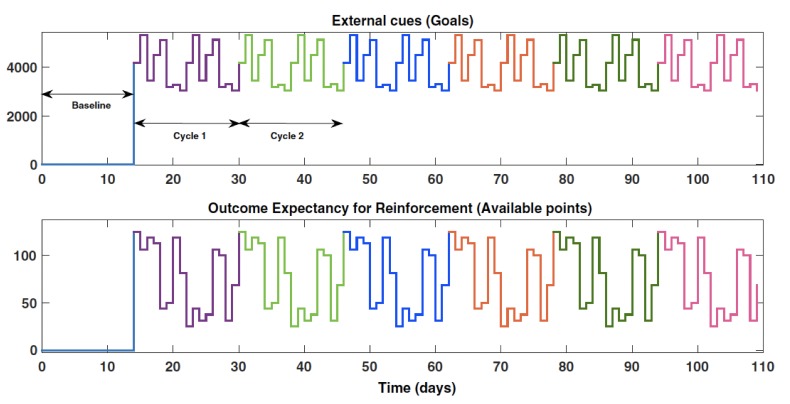
System identification open loop experiment for Just Walk. These two signals were designed a priori using a pseudorandom signal design strategy. This strategy enabled specification of repeated 16-day cycles (delineated as different colors), which allows for robust data for estimation and validation of dynamical models.

A full description on the design of the study is beyond the scope of this paper but has been described elsewhere, which includes concrete strategies for achieving the equivalent of “power” calculations for an open loop system identification experiment [89,121,125]. In brief, our study design involved the pseudorandom suggestion of daily step goals and expected points one could receive if they met their goals as defined in repeated 16-day cycles (Figure 4). On the basis of analyses that are akin to power calculations but for system identification, we determined the need for a minimum of five cycles [121]. Furthermore, the use of 16-day cycles (Figure 4) minimized the risk of possible aliasing with day of the week (which would have occurred with 14-day cycles).

**Figure 5:**
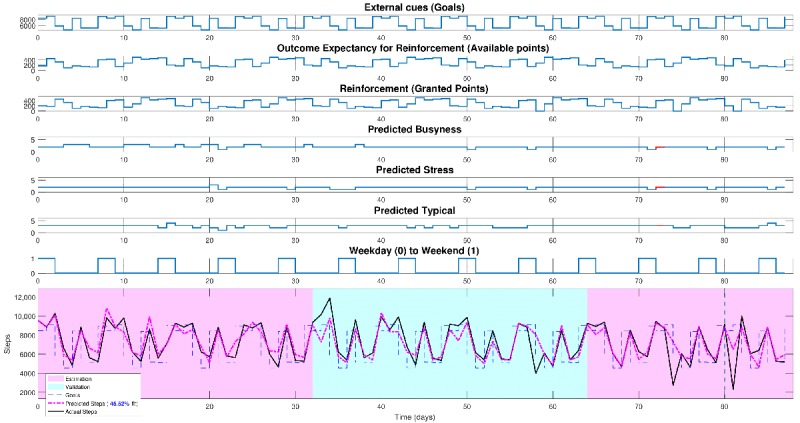
Visualization from one participant from Auto-Regressive Dynamical Modeling.

A full discussion on the analyses and results are beyond the scope of this paper, but interested readers can find more information at [22,89]. In brief, our preliminary analyses on estimating and validating a dynamical model for each person were encouraging both for preliminary dynamical models and the selection of tailoring variables for each person [22,89]. These models produce dynamic daily predictions of steps relative to actual steps (see Figure 5, which visualizes this for one participant). Specifically, Figure 5 visualizes the dynamic interrelationship between the key variables that could be valuable for predicting steps. In this context, this included goals; available points, if points were provided (ie, goal attainment the previous day); a person’s self-reported prediction on how busy and stressed they will be; their prediction on how typical their day will be; and if it is a weekend or weekday. The bottom portion illustrates the predicted steps (pink line) relative to actual steps (black) and suggested goals (dotted blue line). Light pink zones represent cycles that were used for estimation in this particular model, and blue represent validation cycles. Model fit for this participant was 46%, which, based on Cohen’s conventions for multiple regression, would represent a large effect in terms of percent variance explained.

Using percentage model fit as a benchmark, we conducted data-driven analyses to support optimization of the dynamical model that conceptually maps on roughly to reliability and validity. In this case, reliability and validity are estimated for our dynamical models for predicting human behavior (as indicated by model fit) and, by extension, the selection of tailoring variables. For every individual, we conducted an exhaustive search of potential variations of predictors (eg, only our manipulated inputs or up to four additional endogenous or exogenous variables as plausible tailoring variables) using an ARX approach. In line with the leave-one-out approach commonly used when cross-validating models such as PA estimation via accelerometers [126], we carried out estimation or validation using every cycle from our five-cycle system identification study as both estimation and validation data.

For selection of the model and, thus, the tailoring variables to use for each person, we chose to use multiple criteria with the first three reflective of issues of reliability [22,89] and the last more reflective of validity. We chose these criteria to increase the likelihood of finding individualized models that are reliable and, thus, are likely to remain true and appropriate outside of the current data and valid, thus predictive and useful within an eventual controller. We combined them into an approach that penalized models that did not perform as well on these dimensions. Different weights (w) were assigned to four characteristics that affect model consistency and reliability: (1) overall highest fit (w=2), with a higher penalty for lower fits; (2) cross-correlations between inputs (w=2), with higher penalty for inputs with high cross-correlation coefficients; (3) distance of the overall highest fit from the mean fit (mean % fit for all cycle combinations, for each input combination; w=1), with a higher penalty for larger differences; and (4) SD across models run for each participant (% fit for all cycle combinations, for each input combination; w=1), with higher penalty for higher variances. These weights were used to define and select models that were the best estimate in terms of reliability and validity.

We then turned to good enough validity. As these analyses are a variation of multiple linear regression, and the model fit estimate is analogous to r^2^, we chose Cohen’s conventions of explaining 3% of variance as a small effect, 13% as a medium effect, and 26% as a large effect [127]. Although there is no clear definition on good enough for individualization purposes, as, to the best of our knowledge, we are the first to do this, we chose to use the 13% medium effect as our a priori good enough marker for our best model selected for each participant. We also chose a minimum of 50% of our sample to meet this medium effect explained marker as good enough across.

Note, however, that further validity testing related to individualization is possible and a core target of the more definitive optimization trial, the closed loop experiment (see below). Furthermore, we also fully acknowledge that our approach is only one of many (see Discussion). The overall average model fit (estimation and validation data) for all participants combined was 19.2% (SD 9.25). The range was 6.3% to 46%. Using Cohen’s conventions, 20 out of 20 participants met the small effect threshold of explaining 3% of variance, 16 out of 20 met the 13% medium effect level, and 2 out of 20 met the 26% large effect level. On the basis of this, we achieved our good enough target of explained variance for individuals, thus justifying subsequent steps. Although it is unclear what the minimal levels are needed for establishing robust individualization based solely on this, it does provide a preliminary indication of the ability to make distinctions between people in terms of tailoring variables. For example, using the medium effect as a minimal threshold, our approach produced meaningful individualized models for 80% of our sample [22]. From the perspective of pilot testing, we contend that this is likely an adequate target for accounting for individual differences compared with current best practice, though future work is needed to properly specify benchmarks for individualization (see Discussion).

In terms of tailoring variable selection, different tailoring variables were identified for different people [22]. In particular, the most common model included weekend or weekday as the only tailoring variable for 25% of our 20 participants. This model corresponds with the tailoring variable that would likely have been selected when using an aggregate mixed model across participants. Following this, perceived “typicality” of a day and weekend or weekday were the combined tailoring variables selected for 20% of our sample. The rest of participants had different tailoring variables selected. Put differently, if the aggregate model were used for tailoring variable selection, which would be the norm for most methods currently used related to optimizing adaptive interventions, it would have likely selected the inappropriate tailoring variable(s) for 75% of our sample with reliable models [22]. These results point both to the potential for control engineering approaches in individualized tailoring variable selection and for the need for this type of approach.

As of this writing, the team is conducting semiphysical modeling [128] to test our SCT structure (Figure 2). As stated before, the initial models we have already produced use an ARX approach that does not incorporate prior knowledge related to model structure or theorized dynamics [22,89]. As of this writing, we are using these models as our comparators for our SCT model. On the basis of theory, we should get improved model fits when we incorporate the elements of the model we specified, such as model structure, theorized dynamics, etc. If model fits do not improve, it is indicative that our theorized dynamical model structure provides no additional benefit beyond what we would have learned from the data alone for each individual. This is important to highlight as this is a second mechanism for supporting data-driven optimization related to individualization. This time though, the optimization is focused on optimizing the model structure and other theorized prior knowledge.

As this example illustrates, a great deal of valuable insights about human behavior and outcomes can be gleaned from system identification experiments. As this example also illustrates, this step can generate meaningful scientific insights as a mechanism for doing rigorous theory testing that is grounded in an individual first and then can be generalized if similar model structures are found, what we previously called data-driven case studies [124].

### Step 4: Design the Controller, Including Optimization Criteria

The next step is to design the controller. This step is essential as it is the mechanism whereby prior insights can be translated into actionable dynamic decision rules (ie, the controller) for guiding an adaptive intervention. The key steps in this process include defining optimization criteria (eg, set-points), constraints of the controller (eg, clinical constraints of the intervention), and, for more complex controllers, alternative strategies the controller could use to maintain robustness to factors such as a person’s changing responsivity to an intervention. Creation of these is often supported via all of the prior work done (eg, dynamical model, intervention and outcome specification, and system identification experiment), as well as additional simulation studies specifically focused on the robustness of the controller. These steps are done in any type of controller, including those that are not model-based.

In terms of controller design, the central focus of controller design is to define the targeted right balance between performance and robustness. Within a controller, strategies for supporting performance largely revolve around the quality of the previous steps. In particular, performance is improved when potent interventions and predictive models are available to be used by the controller. The prior work provides a foundation for anticipated performance of the intervention options and value of the dynamical model for making predictions. Strategies for maintaining robustness can be devised to help manage and mitigate these risks, which tends to be the larger focus of the controller design for this step. For interested readers, see our more detailed papers formulating our controller [50,129,130] and our strategies for facilitating robustness.

In terms of key tasks, the type of controller being targeted must be defined (eg, model-based or nonmodel based); several parameters for the controller must be defined, including the optimization criteria, constraints, and strategies for achieving robustness; and finally, simulation studies can be conducted to examine anticipated issues of robustness. The optimization criteria can be thought of as a definition of success that can be operationalized based on a measurable outcome variable. Constraints are the parameters that define what is feasible or appropriate within a given domain, such as what is safe, appropriate, or clinically viable. Finally, there are a wide range of strategies for supporting robustness. These assertions can be examined via simulation studies [129,130]. Specifically, control engineering includes methods for simulating plausible responses of controllers within different scenarios and contexts. This is valuable as it enables stress testing assumptions about the problem before recruiting participants.

**Figure 6:**
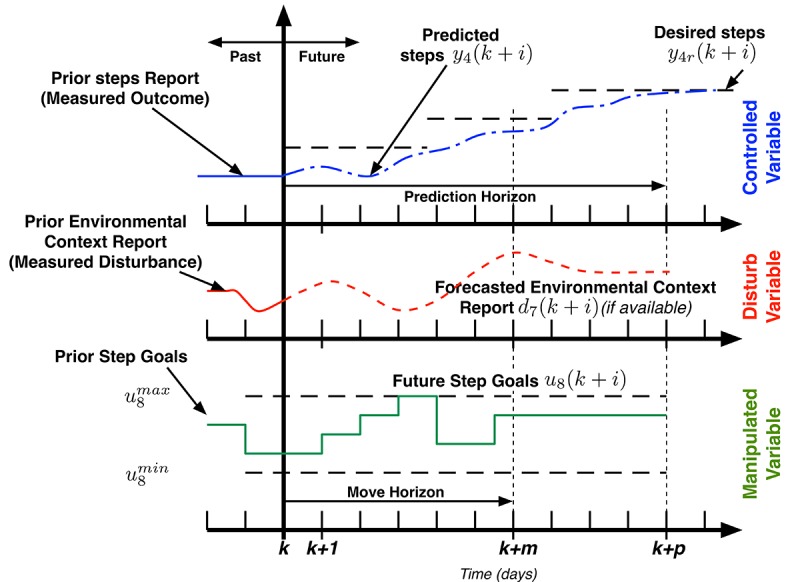
Model-predictive controller “Receding Horizon” strategy. The model predictive controller visualized here is simplified to include only one controlled variable (desired daily steps), one input (ie, goals), and one disturbance (ie, environmental context). Controller moves (ie, goals) are calculated over a horizon, and only the first control move calculated is implemented. The entire procedure is repeated at the next assessment period and continues until the end of intervention.

Within the *Just Walk* example, the controller we chose to use is a hybrid model-predictive controller [131] that can be visualized in terms of its logic for decision making, as shown in Figure 6. As the broad goal of *Just Walk* is to help individuals meet and maintain national guidelines, we set our first optimization criterion when a person is in a state of initiating in more PA up to guidelines of 10,000 steps/day but then also included a less stringent secondary criterion of +3000 steps/day from their baseline step levels based on prior work on anticipated performance of adaptive PA interventions [132]. Prior work has illustrated that +3000 steps corresponds to approximately 30 min/day of moderate intensity activity and, thus, is still a meaningful behavioral target that has the likelihood of reducing disease risk [56,57]. We also included a second optimization criterion for those that meet guidelines and, thus, move to fostering maintenance. Once a person meets guidelines, the system reduces the total number of interactions, including goals suggested and points provided, with the target of reducing interventions to 0 except continued monitoring via the wearable device. We also established clinical constraints, including not changing suggested goals drastically (eg, by more than 4000 steps) from one day to the next. Finally, we have also formulated a variety of secondary strategies the system could take to maintain robustness in case known issues such as reduced adherence are observed.

In terms of how our controller works, the model-predictive controller forecasts changes in outcomes (ie, steps, intervention adherence) over time to determine an error projection that reflects current and expected deviations from the optimization criterion of 10,000 steps/day or +3000 steps/day from baseline. On the basis of this error projection, a real-time optimization algorithm chooses the sequence of future control actions (eg, adjusts step goal, points, and other factors) that minimizes the difference between the set-point (eg, 10,000 steps) and current steps.

The optimization problem is solved for each day considering a prediction time to obtain a predicted optimal step goal suggestion for each decision point. The first recommendation is provided, and the process repeats at each decision point. The model-predictive control strategy continually reevaluates the quality of the previous day’s predictions on what was actually observed. The information can be incorporated into the model-predictive control algorithm, particularly if there are alternative strategies the controller might take based on changing observations for maintaining robustness.

**Figure 7:**
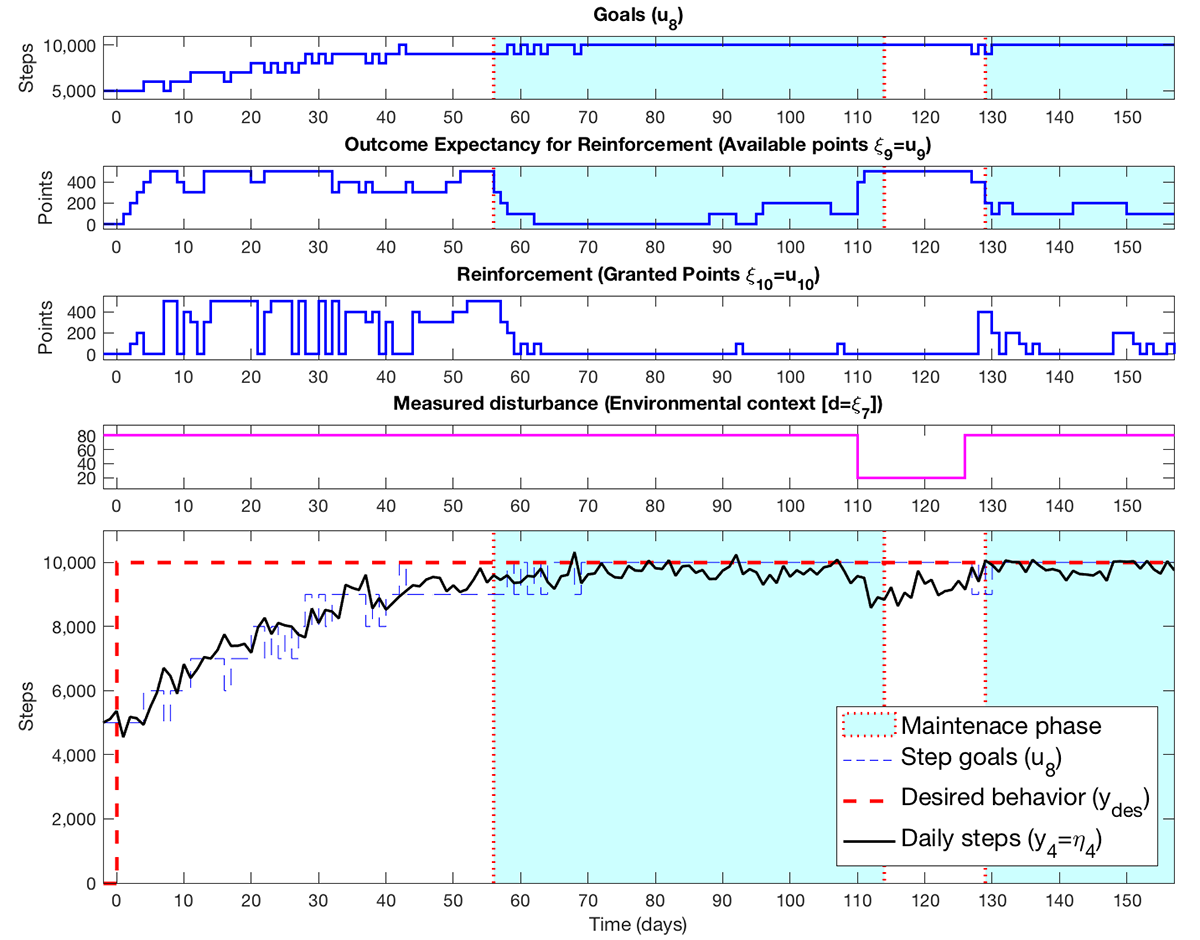
Controller simulation.

We have conducted simulation studies to stress test the design of our controller. Figure 7 is a visualization of one of the simulations we ran for tests of robustness; in this case, the controller’s responsivity to a person experiencing an external disturbance (eg, getting sick). This figure represents a simulation study examining how our controller may respond, in this context, to a major unmeasured environment disturbance. As can be seen, the controller facilitates a gradual increase in steps over time using varying points. When the set-point levels have been reached, the controller switches to a maintenance phase that includes reduced suggestion of step goals (ie, last suggestion would be to maintain 10,000 steps) and reduced use of expected points for meeting the goals (ie, an expanding reinforcement schedule). As the simulation illustrates, the system would strive toward less interaction but be responsive to a person’s steps falling below the set-point level to reactive initiate-phase suggestions (see day 112). For more information on the simulation work we have conducted for our controller see [129,130].

### Step 5: Conduct a Control Optimization Trial

This is the key step for unpacking complex adaptive interventions via control systems engineering methods. This step can provide insights about *how, when, where*, and *for whom* each element functions to produce the desired effect and thus, is the essential strategy for unpacking a complex adaptive intervention and testing its elements. This step should thus, be done whenever the goal is to optimize an adaptive intervention via control systems engineering methods (as opposed to the other plausible adaptive intervention optimization trials). As highlighted above, this is appropriate for the type of problem that has the attributes described in Textboxes 1 and 2.

The key tasks of this step include clear definition of the elements of the adaptive intervention, the design of subexperiments (eg, open loop system identification and closed loop experimentation) and data analysis plan to test the elements, and conducting the trial and the analyses.

As already highlighted, the key elements of an adaptive intervention include the decision points, tailoring variables (or, in this case, dynamical models), decision rules (or, in this case, the controllers), and, of course, the intervention options themselves and the meaningful proximal outcomes the intervention options target. In terms of decision points, these are often defined based on clinical intuition, such as the case in *Just Walk*, whereby our decision point was each morning. These can be tested via control engineering methods as they can be formulated, themselves, as decision rules for guiding just-in-time adaptive interventions, but that point is beyond the scope of this tutorial (and, arguably, MRT is likely more appropriate). As highlighted in step 3, system identification, particularly open loop experimentation, is a rigorous approach for optimizing the tailoring variables or dynamical models for each person and thus, a strategy for optimizing individualization. In terms of the decision rules or controllers, closed loop experimentation is the method to use to test them.

As highlighted in the introduction, it is common in control systems engineering to include multiple experiments provided sequentially, over time, to the same system (ie, person in this context). The key, from a design standpoint of the subexperiments, is to think through what is clinically appropriate or feasible and also what the logical progression is in terms of the likely changes that will occur within the target individual. In terms of the data analytic plan, as with system identification, there is a wealth of analytic strategies that are available, largely within MATLAB, for conducting the analyses. Much of the testing of controllers is actually built into the controllers themselves as, ultimately, they are mathematical equations seeking to minimize error while accounting for noise and other unknown issues. Controllers, thus, engage in self-testing relative to optimization criteria. The key advantage here of self-correction is also arguably a weakness, as this work hinges on the quality of the optimization criteria (a point we return to in the Discussion). A full description of the type of analyses that can be done and the many ways in which to design effective exploration or exploitation is beyond the scope of this introductory text, but interested readers should examine here [104]. Similarly, a full description of analyses for robustness testing is also beyond the scope, but readers can learn more here [43].

**Figure 8:**
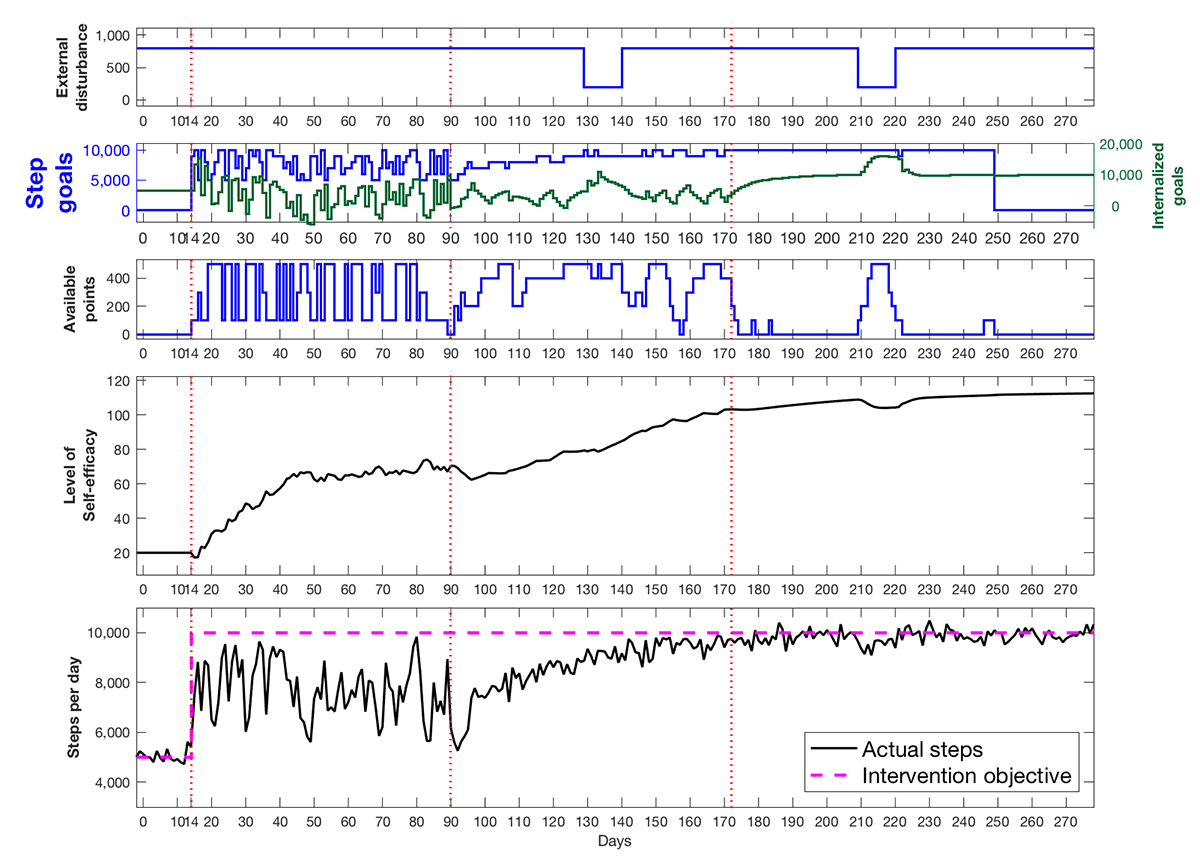
Control optimization trial for Just Walk.

Returning to our *Just Walk* example, we have designed a control optimization trial with four phases specifically designed to test key elements of our adaptive intervention (see Figure 8). The figure is a simulated time series of one participant taking part in our control optimization trial. Row 1 simulates “disturbances” such as getting sick to illustrate how the controller might react (eg, increase points or lower goals). There are four phases divided by the red vertical lines. Phase 1 is an initial measurement only, baseline period, which provides a grounding of the person’s current activity. Phase 2 is an “open-loop” system identification experiment, similar to the study in step 3, whereby step goals (row 2) and points (row 3) are systematically “excited” to enable generation of individualized dynamical models. This phase enables estimating or validating our dynamical model and individualized tailoring variable selection as per our prior study. In phase 3, the model-predictive controller uses those dynamical models to make intervention option decisions to foster initiation of PA towards PA guidelines (row 5) and increased self-efficacy (row 4). During phase 3, the model-predictive controller will strive for appropriate targets for our at-risk group (ie, 10,000 steps/day on average or, if a person does not achieve 10,000 steps/day during initiation, then 3000 steps/day above the person’s baseline median steps). Phase 4 focuses on testing the controller’s decision rules for maintenance (eg, see reduced points provided in row 3). Specifically, we will optimize our approach for providing as minimal support as possible while a person maintains set-point targets.

With this experiment completed, we will be able to systematically test and optimize core elements of our adaptive intervention. In particular, our open loop system identification portion enables data-driven optimization for individualized dynamical models and selecting individualized tailoring variables as described above. Unlike the above work, the final definition of success, which is a person maintaining targeted step levels, will be available and, thus, can be used to define percentage model fits that are, indeed, good enough for individualization purposes. Our closed loop subexperiments allow us to optimize our controller’s ability to achieve set-point targets for each individual for each state, including initiation, maintenance, and possible relapses. We can judge success or failure relative to our optimization criteria (eg, 10,000 steps/day).

Furthermore, we can also produce aggregate (also called nomothetic) information across the sample of participants. Specifically, another optimization check involves comparison of the percentage of our sample that achieves our maintenance targets relative to current best practice PA interventions that appear to produce maintenance targets for approximately 50% of their samples [82,84]. Using previous work as a referent, we can establish the plausibility that our approach is comparable with current best practices if 50% of participants meet our set-point target and exceed current best practices if a higher percentage of our sample achieves our set-point targets. Thus, the control optimization trial can enable both case-by-case (ie, idiographic) optimization for individualization (ie, meeting minimal model fits) and adaptation (amount of time within the desired set-point range across the intervention) and nomothetic optimization (ie, percent of participants meeting target thresholds). This multi-criteria optimization fits with the multiple elements within an adaptive intervention. Furthermore, the study is highly efficient as these elements can all be systematically studied within a single study and, indeed for most of our criteria, on a case-by-case basis.

## Discussion

### Summary

Control systems engineering is a rich discipline that has strategies mHealth researchers and practitioners can use for optimizing elements of adaptive interventions. It is particularly well matched to problems that (1) are dynamic, (2) have useful dynamic interventions available, (3) have an outcome measure that can be measured with sufficient temporal density over an extended time period, and (4) have desirable states for the target outcomes that can be defined as the optimization criteria on a case-by-case basis. There are five suggested (though not necessarily all required) steps for optimizing an adaptive intervention via control engineering: (1) derive a preliminary dynamical model, (2) select intervention options, (3) conduct a system identification open loop experiment, (4) design the controller and optimization criteria, and (5) conduct a control systems optimization trial. This approach holds great promise for expanding the potential of adaptive interventions. This is because control engineering provides a wide range of approaches to systematically unpack and test or optimize the various elements of adaptive interventions both on a case-by-case or idiographic and an aggregate or nomothetic level.

### Connections to Multiphase Optimization Strategy

These steps map on to the MOST framework [28]. Within the preparation phase of MOST, the four suggested steps include the following: (1) develop a conceptual model; (2) develop intervention components; (3) if necessary, pilot test the intervention components; and (4) define the optimization criteria. These steps map on to steps 1 to 4 of the process we delineate but with slight variations based on the requirements for control engineering. An essential difference is step 3, because system identification is valuable not only in preparation for an adaptive intervention (and thus mimics the purpose of step 3 of MOST) but also for theory testing. Thus, it should not necessarily be thought of as pilot testing for the intervention but instead as a valuable scientific pursuit in and of itself. Within MOST, the optimization phase involves conducting an optimization trial, such as a factorial design. One could view system identification experiments, thus, as a form of an optimization trial. That said, the control optimization trial (step 5 in our analogous process) is directly parallel to other optimization trials, as the goal of the trial is primarily on optimizing the intervention, whereas system identification is more focused on theory testing and, thus, not as clearly similar to the optimization trials. If there is interest in seeing if this controller performs better than current standard of care, then the final step of MOST, evaluation via an RCT, can occur. Specifically, if the controller meets the threshold of the optimization criteria, the evaluation phase can then proceed whereby the control-driven intervention can be evaluated relative to a meaningful comparator (eg, current standard of care complex intervention [30]). If, however, the goal is to develop modules that are repurposable, self-contained intervention components (ie, components designed to function separately), then another plausible approach would be to modularize this work for other use cases, as delineated in agile science [103,106].

Beyond the steps, there is also synergy between MOST and control engineering principles. A central focus of MOST is efficiency, including the use of efficient experimental designs and grounding research in real-world constraints related to implementation with the long-term goal of facilitating more efficient and robust knowledge accumulation across studies. Continuous optimization is the second common principle that emphasizes the logic of a continual, iterative process related to further improving and refining behavioral interventions. Control engineering shares these principles of efficiency and continuous optimization. Overall, our work fits well with MOST and current trends in mHealth and the science of behavior change [133].

### Added Considerations Within Control Systems Engineering

As highlighted already, control engineering practices include the principle of triangulation [134]. Unlike the concept of a definitive trial [134], the logic of triangulation (sometimes also called consilience [135]), involves the use of multiple methods and approaches to synergistically study a problem. The basic logic is that every method comes with inherent strengths and weaknesses. When different methods with different strengths and weaknesses point in a common direction, confidence in the assertion increases. Just like how neuropsychologists look for patterns across neurocognitive tests instead of relying on one test, control engineers use a wide range of methods that each have strengths and limitations for iteratively optimizing dynamical models and controllers. This is illustrated in our detailed discussion about a control optimization trial and the many ways in which it can be defined and operationalized via mixed use of open loop system identification experiments, closed loop tests, and robustness testing. If multiple methods and criteria point in a similar direction, then, one can have increased confidence that the overall system is working. Furthermore, if the different tests are not providing consistent results, then the discrepancies can often be used to better understand which elements of the complex intervention are likely inadequate, thus supporting optimization.

These methods are designed to understand and support better prediction and decision making for a given individual. Although we have highlighted the strengths of this approach, there are inherent weaknesses. For example, one potential trade-off exists related to the optimization criteria. If the optimization criteria that are chosen are not meaningful, then even if the controller achieves success (ie, optimization criteria are met), then nothing clinically meaningful has been achieved. This can be mitigated, of course, with optimization criteria that are grounded in clear evidence showing that they are clinically meaningful as is the case within *Just Walk*. Note that this problem is not unique to control systems engineering. It exists within other methods including RCTs, but with RCTs, it manifests via the control condition chosen [136]. In brief, when a poor control condition is chosen, a statistically significant difference may be found (ie, success for this method), but that does not necessarily equate to a meaningful result. To put it more colloquially, one could compare a bad intervention and use a worse intervention as a control, run a trial, and find that bad is better than worse. Unfortunately, the end result is still a bad intervention. Regardless of methods, it is essential to have clarity on what success means, in terms of real-world utility, as is argued in agile science [103,106].

A second major trade-off of control engineering and, indeed, any idiographic approach, is the undervaluing of generalizability to other individuals and contexts. This establishes the need for other methods that are better at balancing this idiographic emphasis with more of a nomothetic emphasis, such as RCTs. With that said, generalization to other individuals and contexts can feasibly occur via a different pathway toward generalizability knowledge, namely, the generalizability concept of causal explanatory models [137]. Shadish et al [137], in their formulation on a theory of generalization, highlighted the concept of causal explanatory models, which are mechanistic models that not only define if there is causal effect (what they called a causal descriptive model and what is produced by an RCT) but *how* the effect occurs, mechanistically. Arguably, dynamical modeling, particularly when robust semiphysical models can be validated, move in the direction of causal explanatory models and, thus, can feasibly aid in improving mechanistic understanding of a phenomenon and, thus, produce generalizable knowledge.

Returning to the concept of triangulation, an RCT can balance out the weaknesses of control engineering methods. As illustrated in the introduction and optimization section, RCTs compromise on providing insights about how, when, where, and for whom a given intervention element works, in the pursuit of stronger internal validity at the intervention package level and also increased external validity in terms of statistical claims of generalizability to the population the sample is conceptually drawn from. As the control optimization trial is an inherently n-of-1 study design, it enables the possibility of it being embedded within an RCT as the intervention arm. This possibility enables a highly efficient way of conducting multiple tests within a single trial that is squarely grounded in the philosophical logic of triangulation, as one trial can test intervention elements and also compare the package to another package. Indeed, including a control condition as a comparison with a control systems optimization trial is, arguably, a highly efficiently rigorous approach to test an adaptive intervention [103].

We emphasize triangulation as we see this as well matched to the complexity of adaptive interventions and possibly behavioral interventions more generally, even outside of the domain of control systems engineering. It is the cornerstone of our key thesis that adaptive interventions are more likely to succeed if its elements can be iteratively improved via optimization. In particular, the fact that there are so many elements within an adaptive intervention (eg, intervention components, decision points, tailoring variables, and decision rules) establishes the need for triangulation. This fits with discussions in psychology, such as the need for a pluralistic approach to causality [134].

### Implications and Future Work

As articulated elsewhere, advancements in digital technologies are rapidly converging to enable a new era in the understanding of human behavior [13,18,138]. A central argument made elsewhere is that the time is right for health and behavioral sciences to reexamine their experimental and analytic strategies [13,18,138]. Although there is great opportunity for a variety of other methods, health and behavioral scientists should more carefully consider control systems engineering. Not only is the time right, from a technical standpoint, but very classic work in psychological science engaged within control theory; thus suggesting that this is really a return to classical roots in psychological science [139-142]. Conceptually, there are many reasons to believe that control systems engineering could be a foundational class of methods behavioral and health scientists could use to improve impact, particularly related to individualized mHealth interventions. Of course, this requires far more research and empirical work before any firm conclusions can be drawn on the potential.

In terms of limitations and future work, more work is needed to clearly evaluate the utility of this approach relative to other methods. For example, the current method used in MOST for optimizing a static intervention is a factorial trial, and SMART and MRT are proposed for adaptive interventions. One valuable test to be conducted is comparison of an optimized intervention to an intervention that was not optimized using these methods. This comparison can be made using an RCT. As the control optimization trial is an inherently n-of-1 method, it is possible to compare the control optimization trial, as a proxy of an optimized intervention, with a control condition that lacks control engineering features. A trial such as this would provide insights on the plausible added value optimization via control engineering may produce relative to more traditional approaches for intervention development whereby the elements are not optimized but, instead, the elements of the intervention are defined based on prior aggregate evidence, user-centered research, and theory.

Building on this point, future work should focus on providing greater clarity on when to use which method for optimizing static and adaptive interventions. As one possible formulation on this, SMART appears useful when the goal is the selection of a progression of decisions to make with relatively infrequent adaptation (eg, once every few months) and with well-specified if-then decision rules. As such, SMART might be particularly valuable within clinical practice. MRT appears particularly valuable for just-in-time adaptive interventions. We argue that control systems engineering methods are likely particularly valuable when the goal is to facilitate a more long-term trajectory of change, such as gradually increasing a target behavior whereby achievement of a desired state cannot happen immediately (eg from 6000 steps/day to 10,000 steps/day or a 5% reduction in weight) but, instead, requires slow progression and building up of skills. Similarly, control engineering methods can also be valuable for facilitating maintenance of a targeted set-point by facilitating small adjustments and provision of support in sort of stepped-care framework. Although we think these general principles are correct conceptually, future empirical work is needed to explore the strengths and limitations of these approaches and the assertions made on when to use which method.

More work related to establishing meaning optimization criteria is needed. This work hinges on well-specified definitions of success that are clinically and practically meaningful but that is not necessarily always available for all elements of an adaptive intervention. For example, we established our model fit estimates as good enough for individualization based on Cohen’s work [127]. We fully recognize that this is an extension and thus may not be appropriate. Future work is needed to think clearly through what good enough optimization is for elements and the adaptive interventions overall.

Finally, future work should further explore if and, if so, how to integrate the logic of triangulation more actively within the development of mHealth interventions. As highlighted before, there is already research starting in this domain but, future research that provides scaffolding for health and behavioral scientists to work through this more complex approach to the design, optimization, and evaluation of interventions could be valuable. We have started this process through the articulation of agile science [103], but further work is needed. Finally, if control engineering does prove valuable, there will be a need for more interdisciplinary training between control engineers and psychologists.

### Conclusions

In sum, mHealth is well poised to take advantage of control engineering methods for the optimization of adaptive interventions. The time is now for health and behavioral scientists to more closely examine control engineering methods. If the approach proves valuable for health problems, new partnerships should be forged between health and behavioral sciences and control systems engineers in the design, optimization, and evaluation of adaptive interventions.

## Abbreviations

ARX: Auto-regressive model with eXogenous Input
mHealth: mobile health
MOST: multiphase optimization strategy
MRT: microrandomization trial
PA: physical activity
PID: Proportional-Integral-Derivative
RCT: randomized controlled trial
RL: reinforcement learning
TTM: Transtheoretical Model
SCT: Social Cognitive Theory
SMART: sequential multiple assignment randomized trial
